# ICON 2020 Supplement

**DOI:** 10.12669/pjms.36.ICON-Suppl.1729

**Published:** 2020-01

**Authors:** Saima Saeed, Qurat ul Ain Shaikh

**Affiliations:** Guest Editors Scientific Committee ICON 2020

The Indus Health Network (IHN) is becoming a major player in Pakistan’s healthcare system. With expanding hospitals, rehabilitation centres, blood banks and public health programs, it provides free-of-cost, accessible, high quality healthcare without discrimination to increasing numbers of ordinary people. ICON, the biennial medical conference hosted by IHN, is a natural beneficiary of this success. It is now a leading platform for sharing excellence in healthcare and research with internationally renowned delegates and a multidisciplinary audience. ICON 2020, the fifth of its series, has an opportunity to go one step further.

In this month’s edition of the Pakistan Journal of Medical Sciences, we are delighted to share and highlight biomedical research at IHN. Distinctly translational and collaborative, IHN supports research via the Indus Hospital Research Centre (IHRC), from study design to publication through to initiating novel service delivery application. The scientific, clinical and programmatic advantage of sharing knowledge and experience, be it rare case reports to trials with international collaborators, is thus at the heart of this first ever publication. It aims to consolidate ethical scientific enquiry at IHN with emphasis on multidisciplinary and thematic research to bridge gaps in knowledge and practice.

It is more than serendipitous that this supplement was organised to coincide with ICON 2020, themed “Building Bridges for Better Healthcare.” From the onset, the Scientific Committee approached the “Herculean” task with a predetermined process. A call for papers was given in March 2019 and kept open until mid-May 2019. In this short time, a tremendous response of 69 articles was received. After the initial administrative shortlist by the Committee, 44 articles were sent for double blind peer review. Each article had two independent reviewers: a national or international subject matter expert and a research professional (biostatistician or epidemiologist) from IHRC. Third reviews were arranged on specific peer reviewer request. Reviews were strictly confidential and conducted on pre-designed scoring proformas for uniformity. They were shared with authors to guide amendments until peer reviewer’s satisfaction. Submissions then underwent internal editorial review for final selections. In all, 20 manuscripts were chosen for publication, blending 11 original research papers, one review article, a case series, two case reports, two letters to the Editor and a section on continuing medical education.

Authors from over 14 medical specialties have come together to create this unique issue. Inevitably some high quality research could not be published due to space constraints or inconsistency with our envisioned theme and purpose. Nonetheless, we congratulate all submitting authors for their efforts irrespective of final outcome. We are grateful to all the reviewers for their time and expertise, indebted to administrative staff who worked behind the scenes and appreciative of the ICON Organizing Committee’s support. We also wish to give special thanks to the Pakistan Journal of Medical Sciences for the publication in their highly reputed journal and guidance in the process.

Welcome to ICON 2020! We hope to see you at the Marriott Hotel, Karachi, January 17-19, 2020.

